# Supplementary material for: Cross-culturally adapted psychological interventions for the treatment of depression and/or anxiety among young people: A scoping review
Source: PLoS One. 2023 Oct 25;18(10):e0290653. doi: 10.1371/journal.pone.0290653 (PMC10599551; doi:10.1371/journal.pone.0290653)
Supplement: S1 File — (DOCX) [file pone.0290653.s001.docx]

S1 File

Search strategy Ovid MEDLINE

1 Behavio* activation.ti,ab.

2 (behavio* adj2 (intervention* or therap* or treatment* or psychotherap* or psychotherap*)).ti,ab.

3 Behavior Therapy/

4 Cognitive Therapy/

5 self monitor*.ti,ab.

6 (Activit* adj3 (schedul* or plan* or arrang* or organis* or organiz*)).ti,ab.

7 1 or 2 or 3 or 4 or 5 or 6

8 Depression/

9 exp Depressive Disorder/

10 Depression.ti,ab.

11 Depressive.ti,ab.

12 Depressed.ti,ab.

13 ((low or negative or decreas*) adj2 (mood* or affect)).ti,ab.

14 8 or 9 or 10 or 11 or 12 or 13

15 7 and 14

16 young person*.ti,ab.

17 (child* or schoolchild*).ti,ab.

18 teen*.ti,ab.

19 adoles*.ti,ab.

20 (pre-pubert* or prepubert*).ti,ab.

21 (pre-teen* or preteen*).ti,ab.

22 Child/

23 Adolescent/

24 student*.ti,ab.

25 16 or 17 or 18 or 19 or 20 or 21 or 22 or 23 or 24

26 (cultural* or adapt*).ti,ab.

27 (cross or cultur*).ti,ab.

28 26 or 27

29 15 and 25 and 28
