# Supplementary material for: Cross-culturally adapted psychological interventions for the treatment of depression and/or anxiety among young people: A scoping review
Source: PLoS One. 2023 Oct 25;18(10):e0290653. doi: 10.1371/journal.pone.0290653 (PMC10599551; doi:10.1371/journal.pone.0290653)
Supplement: S3 File — (DOCX) [file pone.0290653.s003.docx]

**S3_ Tables**

**Supplementary Table 3a- 3c**

**Supplementary Table 3a:** The results reported in the Randomized Control Trials

| **Study Author**  **year** | **Measure** | **Intervention group** | | | **Control group** | | | **Change reported** |
| --- | --- | --- | --- | --- | --- | --- | --- | --- |
|  |  | **Pre-treatment (mean, SD) n** | **Post-treatment**  **(End of treatment point) (mean, SD) n** | **Follow-up (mean, SD) n** | **Pre-treatment (mean, SD) n** | **Post-treatment**  **(End of treatment point) (mean, SD) n** | **Follow-up (mean, SD) n** |  |
| **Studies reported both anxiety and depression** | | | | | | | | |
| Alampay 2020 | *STAIC* | *2.11 (0.26) n= 87* | *2.08 (0.29)*  *n=74* | *1.99 (0.32) n=70* | *Handicraft*  *1.99 (0.33) (Control group of making handicraft)*  *n=99* | *1.96 (0.37) n=90* | *1.97 (0.31) n=76* | *No significant changes in anxiety scores* |
|  | SMFQ | 0.74 (0.32)  n=87 | 0.77 (0.39)  n=74 | 0.68 (0.37) (2-month follow-up)  n=70 | 0.72 (0.34)  n=99 | 0.65 (0.36)  n=90 | 0.53 (0.35)  n=76 | Depression scores for the Handicrafts group decreased over time, however the Kamalayan group’s depression score remained stable over time (*b* =− 0.02, *SE* = 0.02, *p=*.24) |
| Ishikawa  2019 | *ADIS CRS* | *Mean (SE)*  *6.31 (0.49) n=26* | *3.08 (0.50)*  *n=25* | *1.43 (0.52) n=24 (6 month)* | *6.72 (0.50) n=25* | *6.00 (0.51)*  *n=24* | *1.57 (0.52)*  *n=24* | *50% (n=13) of those randomised to CBT free of their principal diagnoses at post-treatment compared to 12% (n=3) in the WLC(χ2 (1, N = 51) = 8.55, η2 = 0.17, p<.01)* |
|  | *SCAS* | *37.3 (3.55)* | *28.28 (3.55)* | *20.20 (3.55)* | *41.58 (3.63)* | *35.95 (3.97)* | *18.57 (3.70)* | *No significant difference between the CBT and WLC on child self-reported anxiety.* |
|  | DSRS | 16.85 (1.44) | 14.00 (1.54) | 12.36 (1.47) | 16.74 (1.47) | 16.50 (1.50) | 8.87 (1.54) | Clinician-rated severity and child-reported depression showed improvements in symptoms in the CBT condition compared to WLC(significant improvements of the CDI). All participants with depressive disorders free from their principal diagnosis at 6-month follow-up |
|  | CDI | 18.75 (1.78) | 14.64 (1.75) | 14.40 (1.75) | 18.87 (1.82) | 19.05 (1.86) | 12.61 (1.82) |  |
| Listug-Lunde et al. (2013) | CDI | 21.00 (5.29) n=8 | 14.39 (9.93) n=8 | 14.13 (8.11) n=8 | 20.37 (4.10)  n=8 | 13.25 (9.87)  n=8 | 11.25 (6.41) n=8 | Both groups had significantly lower CDI scores from pre- to post-treatment to 3-months follow-up. There was a significant difference between pre-treatment and post-treatment scores and between pre-treatment and 3-month follow-up scores |
|  | *MASC* | *51.57 (14.44) n=7* | *43.29 (23.01) n=7* | *40.14 (24.14) n=7* | *44.25 (23.93)*  *n=8* | *37.00 (23.34) n=8* | *46.00 (16.94) n=8* | *No significant differences in MASC scores from pre- to post-treatment to 3-months follow-up* |
| Li et al.  2022 | CDI-S | 5.64 (2.63)  n=45 | 5.26 (3.81) n=44 | 6.06 (4.91) n=44 | 4.89 (2.89)  n=42 | 6.19 (4.32) n=40 | 5.71 (4.18)  n=41 | No significant difference in depression scores between groups at post-treatment or follow-up |
|  | *CSAS-S* | *18.41 (4.12) n=45* | *17.87 (4.17) n=44* | *18.42 (4.78) n=44* | *18.65 (3.74)*  *n=42* | *19.99 (4.44)*  *n=40* | *20.09 (4.97)*  *n=41* | *Significantly lower anxiety scores in the intervention group post-treatment but not at follow-up.* |
| Ramdhonee-Dowlet et al. 2021 | *RCADS: anxiety subscales* | *53.22 (19.89)*  *n=50* | *36.50 (9.21) n=50* | *40.44 (12.52) n=50* | *50.10 (22.34)*  *n=50* | *85.88 (11.29) n=50* | *72.48 (22.06) n=48* | *Significantly lower anxiety scores in the intervention group at post-treatment and follow-up* |
|  | RCADS: Depression subscales | 11.16 (6.43) n=50 | 6.14 (2.90) n=50 | 7.06 (3.08) n=50 | 11.10 (6.15) n=50 | 19.10 (2.67) n=50 | 20.36 (2.31) n=48 | Significantly lower depression scores in the intervention group at post-treatment and follow-up. |
| **Studies reported depression** | | | | | | | | |
| Bernal 2019 | CDI | 22.97 (7.72) n=61 |  |  | (Second treatment group CBT alone)  21.68 (7.38) n=60 | Estimate regression coefficient (Standard error)  1.019 (0.071) |  | From pre-treatment to post-treatment, 76.7% of CBT alone and 80.4% of CBT and TEPSI group demonstrated a reliable change in depression scores. At 12-months post-treatment, 69.5% of CBT only and 67.2% of CBT and TEPSI groups remained in remission for MDD. |
|  | CDRS-R | 52.46 (9.13) n=61 |  |  | 52.00 (8.27) n=60 | -1.106 (0.051) |  |  |
| Damra 2014 | CDI | 42.77 (2.49), n=9 | 26.44 (3.84)  n=9 | 27.44 (1.94)  n=9 | 44 (1.87)  n=9 | 43.77 (3.84)  N=nr | 44 (1.87) | Pre, post, and follow-up CDI scores in both groups differed significantly (*F*= 99.617,*df* = 1.247,*p<* .05) (the within-subjects effects results) |
| Rossello and Guillermo Bernal 1999 | CDI (IPT) | 21.21 (7.53)  n=23 | (10.79 (6.51)  n=19 |  | Wait-list control  20.13 (5.99)  n=23 | 15.83 (6.83)  n=18 |  | Both the IPT and CBT groups had statistically lower depression scores at post-treatment compared to the WLC. 77% of IPT group and 67% of CBT group had better outcomes in depressive symptoms at post-treatment compared to WLC (*F*=6.69, *p<*0.01). No statistically significant differences found on the CDI scores between the IPT and CBT groups. |
|  | CDI (CBT) | 20.12 (6.95)  n=25 | 13.28 (7.61)  n=21 |  |  |  |  |  |
| Rossello, Bernal and Medina 2008 | CDI | (IPT group ) 21.52 *(*6.88)  n=29 | 14.62  *(*7.33)  n=28 |  | (CBT group) 22.62 *(*7.16)  n=23 | 12.04  *(*6.98)  n=28 |  | Reductions in CDI scores seen in both IPT and CBT groups. Significantly greater reductions in scores were reported in those randomised to CBT (*F*(1, 107)= 5.96, *p*=.016 compared to IPT. The format of treatment (i.e. group or individual delivery) did not have a significant effect on CDI scores (*F*(1, 107) =1.01, p=.316) |
| Saw, Tam and Bonn 2019 | RADS-2 | 80.20 (4.83) n=10 | 68.90 (10.29) n=10 | 59.80 (14.94) n=10 | 79.10 (3.67) n=10 | 77.80 (1.75) n=10 | 82.30 (4.03) n=10 | Significant decreases in depression scores in the intervention group from pre-intervention to post-intervention and 1-month follow-up. Increase in depressive scores in control group not statistically significant. |
|  | ATQ-Malay | 46.60 (3.62) n=10 | 33.70 (5.54) n=10 | 32.50 (5.15) n=10 | 47.00 (3.94) n=10 | 43.60 (2.63) n=10 | 41.40 (6.62) n=10 | Significantly greater reduction in automatic negative thoughts from pre-intervention to post-intervention maintained at follow-up for intervention group compared to control group. |
| **Study reported anxiety** | | | | | | | | |
| Khan 2020 | *AS* | *33.42 (2.64), n=12* | *25.33 (2.15), n=9* |  | *35.75 (5.03), n=12* | *39.67 (4.23), n=9* |  | *Significant decrease in anxiety sensitivity, overall anxiety (F=5.43, ω2=.44); generalized anxiety (F=5.07, ω2=.43) from pre-to post-assessment in experimental group compared to control group* |
|  | *OA* | *41.58 (5.73)* | *29.33 (12.75)* |  | *44.33 (7.63)* | *45.42 (7.71)* |  |  |
|  | *GAD* | *9.67 (3.02)* | *5.50 (2.68)* |  | *9.08 (2.15)* | *9.33 (3.08)* |  |  |

**Notes.** All anxiety measures in the above table are shown in italics. WLC: Wait-List Control; CBT: Cognitive Behavioural Therapy; IPT: Interpersonal Psychotherapy Treatment. Depression Measures: SMFQ: Short Mood and Feelings Questionnaire; CDI: Children’s Depression Inventory; CDRS-R: Children’s Depression Rating Scale-Revised; DSRS: Depression Self-Rating Scale; RAD-2: Reynolds Adolescent Depression Scale–Second Edition; ATQ-Malay: Automatic Thoughts Questionnaire - Malay version. Anxiety Measures: *STAIC: State-Trait Anxiety Inventory for Children; CRS: Clinical Significance Ratings; ADIS: Anxiety Disorders Interview Schedule for DSM-IV (ADIS); SCAS: Spence Children’s Anxiety Scale; MASC: Multidimensional Anxiety Scale for Children; AS: Anxiety Sensitivity; OA: Overall Anxiety; GAD: Generalized Anxiety Disorder*

**Supplementary Table 3b:** Results reported in the non-randomized trials with a single treatment arm

| **Study Author/year** | **Measure** | **Pre-treatment (mean, SD) n** | **Post-treatment**  **(End of treatment point) (mean, SD) n** | **Follow-up (mean, SD) n** | **Significance** |
| --- | --- | --- | --- | --- | --- |
| *Acarturk 2019* | *SCARED Mean (95% CI)* | *28.2 (20.1, 36.2), n=13* | *19.6 (15.1, 24.1), n=13* | *14.0 (9.2, 18.8), n=13* | *The differences in scores reported across time points were statistically significant (F(1.4, 16.8) = 19.1, p<.001)* |
|  | BDI  Mean (95% CI) | 11.9 (6.8, 17.1), n=13 | 6.5 (4.6, 8.3), n=13 | 5.2 (3.4, 6.9), n=13 (2 month) | The differences reported across time points were statistically significant (*F*(1.2, 14.7) = 8.8, *p<*.01.) |
| Goodkind  2010 | *MASC* | *13.29 (6.50), n= 24* | *9.64 (5.07)*  *n= 20* | *10.34 (7.42) (6 month follow-up)*  *n= 17* | *For anxiety symptoms (total MASC scores), there was a significant linear fixed effect, t(75)=2.15, p<.05, indicating a significant linear decrease in anxiety symptoms of approximately 1 point per 3-month interval.* |
|  | CDI | 16.06 (3.97)  n= 24 | 14.64 (4.52)  n= 20 | 15.44 (8.34)  n= 17 | For depression symptoms (total CDI score), the best fitting model had a fixed effect for linear change that was marginally significant and negative,  *t*(22)=1.98,*p=*.06. indicating a reduction in CDI scores across participants  of approximately .5 point per 3 months. |
| Morsette 2019 | CDI | 14.29 (NR) n=7  15.00 (NR) n=4 | 4.50 (NR)  n=4 | n/a | Three out of four participants completing treatment improved significantly in depressive symptomatology and two out of the four in terms of PTSD symptoms |
|  | *CPSS* | *8.50 (NR), n=4* | *3.50 (NR), n= 4* |  |  |
| Orgiles  2019 | *SCARED*  *Mean (95% CI)* | *27.32 (26.71, 27.93)*  *n=119* | *25.20 (23.58, 26.82)*  *n=112* | *22.55 (20.45, 24.66)*  *n=110*  *(12 months follow-up)* | *After the intervention, children reported significantly lower scores for measures of generalized anxiety (p=.03), and separation anxiety (p= .02) compared to baseline.* |
|  | CDI  Mean (95% CI) | 11.35 (10.85, 11.84) | 9.14 (8.11, 10.18) | 7.77 (6.87, 8.68) | After the intervention, children reported significantly lower scores for measures of depression (*p=* .001) |

**Notes.** All anxiety measures in the above table are shown in italics. Depression Measures: BDI: Beck Depression Inventory; CDI: Children’s Depression Inventory. Anxiety Measures: *CPSS: Child PTSD Symptom Scale; MASC: Multi-dimensional anxiety scale for children; SCARED: Screen for anxiety related emotional disorders.*

**Supplementary Table 3c: results reported in the case studies**

| Study Author  year | **Participant** | **Measure** | **Pre-treatment (mean, SD)** | **Post-treatment**  **(End of treatment point) (mean, SD)** | **last Follow ups (mean, SD)** | **Clinical change** |
| --- | --- | --- | --- | --- | --- | --- |
| Binkley 2017 | **1** | CDI-2 | Not reported | Not reported |  | CDI-2 scores reduced to the normal range across all domains for sessions 2 and 4 At 1-month follow-up call with parent the participant’s mood continued to remain stable and she demonstrated increased engagement.  RCI: *NR* |
| Duarte ́-Ve ́lez, 2010 | **1** | CDRS (R) | 58 | 41 | 28 (15-months) | Participant no longer met criteria for MDD at final session, but continued to meet criteria for a non-specified anxiety disorder and for attention deficit disorder and hyperactivity, predominantly inattentive. The outcomes on symptom scores for depression (CDI and CDRS-R) and dysfunctional thoughts (DAS) from baseline to a 12-months post-treatment showed a notable decrease in depression on both measures from baseline to post-treatment. His DAS scores also decreased substantially from baseline to the 1-year follow-up. RCI: NR |
|  |  | CDI | 27 | 18 | 11 |  |

**Notes.** Depression Measures: Children’s Depression Inventory–Second Edition (CDI-2), Children’s depression rating scale-revised (CDRS-R); Reliable Change Index (RCI)
