# Supplementary material for: Cross-culturally adapted psychological interventions for the treatment of depression and/or anxiety among young people: A scoping review
Source: PLoS One. 2023 Oct 25;18(10):e0290653. doi: 10.1371/journal.pone.0290653 (PMC10599551; doi:10.1371/journal.pone.0290653)
Supplement: Attachment — Submitted filename: Review_Mishu et al., 2023_PLOS ONE_submitted.docx [file pone.0290653.s004.docx]

The authors summarize current research on cross-culturally adapted psychological interventions for (pre-)adolescents with focus on the treatment of symptoms of anxiety and depression. This is a topic of growing importance, especially bearing in mind that global prevalence rates of mental disorders recently sharply increased in youth populations in connection to Covid-19.

However, there are a number of issues concerning the article‘s main focus of interest , the chosen methodology, and the derived conclusions from the data collected that should be clarified before puplication.

Abtract:

- After revision of the article, an adaptation of the abstract‘s content has to follow

Introduction:

- The authors should pay attention for a consequent use of a cultural sensitive and political correct language. Thus, attributions like „brown“, „black“ and „white“ (p.4) should be appropriately replaced. Same applies to „original country“ (p.5) for which the term „country of origin“ is suggested, instead.
- The theory behind culturally adapting a psychological intervention is just briefly described in the introduction. Instead of introducing common frameworks/models (e.g. Bernal & Sáez-Santiago; „surface vs. deep stractuture adaptations“) within the results, it would be helpful for the reader to give more information about ways of adapting an intervention already within the introduction.
- The authors don’t provide information why they decided for preparing a scoping reveiw instead of a systematic review.

Unfortunately, the study’s main purpose is not clear to the reader, yet. From the abstract and the introduction, it reads as if the authors are primarily interested in examining the effectiveness of culturally adapted interventions for adolescents (although, if this ist he case, the intention could be formulated more concisely). However, going through the discussion, the authors mainly focus and give recommendations on the concrete process and different approaches of culturally adapting an intervention, so that the common thread is missing. A revision in the sense of creating a coherent structure is advised.

Methods:

- The authors provide full information on the algorhythm used for their literature research. For the interest of transparency, it would be helpful, however, if they could explain the underlying principles for their search strategy in a few sentences.

Results:

- Consistent use of italic letters for statistical letters (e.g. „M“, „SD“)
- Table 1: Consistent use of bold letters for headings; consistent, equal structure of table cells (e.g. column „setting and sample“ – please provide information on diagnoses and country of implementation for every study – if unknown, this can be noted accordingly); about adding a column mentioning the kind of cross-cultural adaptation process used by each study should be thought
- Table 3 a-c: These tables all are a bit too confusing for the reader. It should be visible on first sight, which numbers are representing levels of anxiety and which levels of depression. Also, significant changes (pre vs. post as well as treatment- vs. control-group) should be detectable directly for the reader. The column „change reported“(3a)/“significance“ (3b) ist very word-heavy. Table 3c can be omitted completely as it provides almost exclusively text content which can be presented within the article’s written results just as well.
- Figure 2: The benefit from this illustration is arguable as the authors desribe the shown adaptation processes in detail within the results.
- In general, the information provided within the tables 3 a-c are repetitive compared to the written results, to a great extend. Due to the large methodological heterogenity of the reviewed studies, the authors chose a narrative synthesis for the presentation of the studies‘ results. However, an abbreviated and focused display of either the written results or the table contents is advisable.
- p. 23: The authors describe having consulted various stakeholders for discussing their preliminary review findings. From the information shared in the current article, it isn’t clear, however, whether the discussion followed any kind of structured frame or was intentionally kept open. Furthermore, the discussion’s goal and thus the benefit for the current article doesn’t really stand out, so far. Concerning the last paragraph on p. 23, it’s not recognizable whether the authors reproduce the stakeholders‘ points or summarize their own thoughts (which would be misplaced within the results).

Discussion:

- As mentioned above, the discussion’s focus is orientated differently than indicated by abstract and introduction. If the study aims to access the effectiveness of culturally adapted psychological interventions, then the authors have to allow way more space for this topic and discuss their findings critically (e.g. efficacy pre/post, efficacy treatment/non-treatment, (dis-)advantages of different types of control groups, etc.).
- The authors‘ remarks concerning the benefits of a participatory approach when designing culturally adapted interventions was enlightening. However, the initial observation that „bottom-up“ interventions tend to be more effective than „top-down“ interventions cannot be derived from the results that are presented. Similar applies to the conclusion about emic and etic dimensions by Rosselló and colleagues (p.27), which isn’t mentioned in the article before and is accepted as fact by the authors without further discussion or referencing other supporting literature
- Results of the stakeholders discussion (if it shall remain within the article) aren’t discussed at all.
