# Supplementary material for: Cross-culturally adapted psychological interventions for the treatment of depression and/or anxiety among young people: A scoping review
Source: PLoS One. 2023 Oct 25;18(10):e0290653. doi: 10.1371/journal.pone.0290653 (PMC10599551; doi:10.1371/journal.pone.0290653)
Supplement: Attachment — Submitted filename: Response to Reviewers.docx [file pone.0290653.s005.docx]

**Response to Reviewers Comments**

**Paper Title:**  Cross-culturally adapted psychological interventions for the treatment of depression and/or anxiety among young people: a scoping review

We would like to thank the peer reviewers for their valuable comments. We are submitting a revised version of the manuscript that addresses the points raised during the review process. Our responses to all comments are presented in the table below.

**Reviewer 1**

| **Comments** | **Response** |
| --- | --- |
| 1.This is a well written and interesting piece of research which highlights the need for depression and/or anxiety interventions delivered to adolescents to be culturally and contextually sound. | Thank you for your comments |
| General comments:  2.While generally well written and intelligible, there are a few grammatical errors in the article which need to be paid attention to. For example, in the Study Participants section, the first sentence does not make sense without a comma after the word ‘studies’. This happens at various occasions, and needs to be corrected, otherwise it is difficult for the reader to understand what the sentence means.  Some abbreviated words are used without their full name in the first use, e.g RCT and USA. USA also needs to be kept for consistency e.g in the discussion, the authors write ‘US’ instead of ‘USA’ which is used earlier in. | We have now added a comma into the ‘study participants’ and have identified and corrected similar errors.  We have now ensured that all abbreviated words are presented in full when first used. We have changed ‘US’ to ‘USA’ in the discussion for consistency. |
| Introduction: |  |
| 3.It would be helpful to define ‘adolescence’ in the introductory section. Also, the authors include studies of those aged 8 to 18 years old. It would be helpful to understand why this age range is included, when adolescence is most commonly defined as 10 to 19 (World Health Organization).  Also, sometimes the word ‘children’ is included, which confuses the reader e.g in the sentence ‘psychological interventions have the potential to improve the mental health of children and adolescents’. I suggest changing the phrasing throughout to young people, as per age criteria and definition, or sticking to adolescents. | As suggested by the reviewer, we have changed the phrasing throughout to ‘young people’. However, when describing referenced studies, we have used the term employed by the study (children and/or adolescents).  To clarify this, we have now added the following sentence into the abstract: ‘defined here as children and adolescents aged between 8-18 years’ and in the introduction. |
| Methods |  |
| 4.The methods section is very detailed and meets the PRISMA-ScR guidelines. It would be useful to include a PRISMA-ScR checklist in the supplement or figures to show where each item has been included: <http://www.prisma-statement.org/documents/PRISMA-ScR-Fillable-Checklist_11Sept2019.pdf> | We have now added the PRISMA-ScR as supplementary file 2. However, the page number will need to be revised when the article will be published as per the journal’s requirement. |
| 5.There needs to be some clarity on the recruitment of stakeholders, where they were sampled from, why they were chosen, which culture/country, and ethics used for adolescent involvement. Where the authors wrote ‘participants shared their perspectives on the need and acceptability of cross-culturally adapted interventions and made recommendations’, this needs more clarity. What interventions? Why are their opinions valid or useful here? | Thank you for your comment. We have added some additional text to the ‘stakeholder consultation’ section. Ethical approval was not required as it was part of Patient and Public involvement (PPI), in the form of consultation and participants were selected from within existing networks.  We clarified, what interventions and why their opinions are useful here:  Alongside the scoping review, we consulted with one adolescent and three parent stakeholders, each of them from an ethnic minority community living in the UK and with lived expertise of depression and/or anxiety (either having experienced this themselves or having looked after someone experiencing this). The purpose of this was to increase our accountability to the members of the communities we are researching, by allowing them the opportunity to dispute or endorse our findings, to add in their own voices and perspectives, and also to help us identify what further research should be prioritised. |
| 6.For the eligibility criteria, what makes a population or study ‘wrong’? This needs to be better phrased. Also, no restriction is placed on language. How many studies were written in a language other than English, and how did you read these? It would also be good to understand and include a sentence on why grey literature was not included (rationale for eligibility criteria is a key feature of the PRISMA-ScR guidelines). There is bias in using western databases to report interventions delivered to non-western populations, which needs some recognition. The authors could be missing key literature | We have now added more clarification into the section ‘excluded studies’:  ‘…,18 were excluded. Reasons for exclusion included: having the wrong population (age group more than 18 years old) (n=9), wrong publication type (conference abstract, review, report, editorial, study protocol, any grey literature) (n=1), not mentioning cultural adaptation/related process/effectiveness (n=7), and unobtainable full text (n=1).’  One study title was written in Arabic but the abstract was written in English. That study was excluded at the abstract screening stage due to not meeting our inclusion criteria.  We have now acknowledged the limitation relating to bias and not including grey literature in the discussion section: ‘We used the main international databases for conducting our search, however, there might be bias in using these databases to report interventions delivered to non-western populations. It was beyond the scope of the study to conduct searches in different regional or country-specific databases and those which included grey literature’. |
| 7.For the data extraction section, it would be useful to define ‘any relevant outcome’, as this is quite vague. | We have clarified what outcome data were included:  ‘any relevant outcome data (depression and/or anxiety)’ |
| **Results:** 8.I suggest putting the results/outcomes from Tables 3a and b into Table 1, rather than lots of different tables, as this allows the reader to have all information of each study in one place. | Thank you for your suggestion. We have moved tables 3a-c (containing detailed outcome reports) into a supplementary file and updated the findings in Table 2. We have chosen not to amalgamate the tables as we feel this will make the table too busy and difficult to follow. |
| **Discussion:** |  |
| 9.The paragraph which says that there is ‘little or no consideration of cost-effectiveness’ of culturally adapted vs. non adapted interventions needs some references. Is there any evidence to support this? | Thank you for your suggestion. This statement came from the findings of our review as none of the 17 identified studies assessed cost-effectiveness, therefore no reference was made. We have added some text into the discussion:  ‘There is little or no consideration in the reviewed studies of the cost-effectiveness of culturally adapted over un-adapted interventions as none of the 17 identified studies attempted to assess cost-effectiveness and this is an area of much needed research’ |
| 10.I suggest including a note that the studies were from a range of different populations and cultures/contexts. Your article groups together a wide range of very different populations, which may oversimplify individual cultural needs and be seen as problematic. | Thank you for your comments. We have now added this point as one of the limitations of the review: ‘Finally, we acknowledge that, in grouping together studies from a wide range of different populations and from different cultures and contexts, there is a risk that we overgeneralise and oversimplify the actual complexity of individual cultural needs’. |
| 11.Section on ‘A participatory approach’ – do we know why three of the seven young people dropped out of the programme? I think there should be some reference to participatory involvement in your stakeholder interviews and results section, not just in the discussion and conclusion. | Many thanks for raising this point. The Morsette et al. (2009) paper mentioned the reasons for why young people dropped out and it was not related to the intervention: *“One participant moved out of the school district, one’s parents removed the student from the program after child protective services were contacted, and one participant encountered serious medical problems and was hospitalized.”*  As the reasons for why the young people dropped out was not related to the interventions, we have removed the sentence- ‘In spite of this, however, three out of seven of the young people dropped out of the programme’ from our manuscript.    In the Results section, we added related text.  In the stakeholder’s section we added that: ‘Based on the feedback of our stakeholder consultations we found that a ‘participatory approach’ by engaging different community level stakeholders in the process of adaptation was highly recommended.’  As suggested, we have added participatory involvement in the results and stakeholder sections, and have revised the related part in the discussion. |

**Reviewer 2:**

Many thanks to the reviewer for their encouraging comments. No improvements were recommended.

**Reviewer 3:**

| **Comments** | **Response** |
| --- | --- |
| Introduction: |  |
| 1.The authors should pay attention for a consequent use of a cultural sensitive and political correct language. Thus, attributions like „brown“, „black“ and „white“ (p.4) should be appropriately replaced. Same applies to „original country“ (p.5) for which the term „country of origin“ is suggested, instead. | Thank you for your suggestion. We originally used these terms as these were used in the original papers that we referenced. However, we have now replaced several words as appropriate. |
| 2.The theory behind culturally adapting a psychological intervention is just briefly described in the introduction. Instead of introducing common frameworks/models (e.g. Bernal & Sáez-Santiago; „surface vs. deep stractuture adaptations“) within the results, it would be helpful for the reader to give more information about ways of adapting an intervention already within the introduction. | Thank you for your suggestion. We have now added a section into the introduction: ‘Different theories and frameworks for the process of cross cultural adaptation have been developed and used. One of the more commonly used frameworks is the ecological validity and cultural sensitivity framework developed by Bernal and Sáez-Santiago (25), where adaptation posits eight dimensions (language, persons, metaphors, content, concepts, goals, methods, and context) to increase the ecological and external validity of a treatment. Some other cultural adaptation processes include surface and deep structure levels adaptation (26,27), the planned adaptation approach (28), and content cultural adaptation (29,30) . |
| 3.The authors don’t provide information why they decided for preparing a scoping reveiw instead of a systematic review. | We adopted a scoping review as this is a more exploratory approach and the research area is still in a preliminary stage and very broad. We now added related text in the Introduction. |
| 4.Unfortunately, the study’s main purpose is not clear to the reader, yet. From the abstract and the introduction, it reads as if the authors are primarily interested in examining the effectiveness of culturally adapted interventions for adolescents (although, if this ist he case, the intention could be formulated more concisely). However, going through the discussion, the authors mainly focus and give recommendations on the concrete process and different approaches of culturally adapting an intervention, so that the common thread is missing. A revision in the sense of creating a coherent structure is advised. | Thank you for this helpful comment. We have now clarified the point and made the following changes.  Objectives:  1. Identify available studies that tested the cross-culturally adapted interventions for the treatment of depression and/or anxiety among young people.  2. to explore the cross-cultural adaptation process and frameworks used for the cultural adaptation.  3. to examine the effectiveness of these adapted interventions in the treatment of depression and/or anxiety disorders among young people.  We have also ensured that these changes are reflected in our abstract. |
| Methods: |  |
| 5.The authors provide full information on the algorhythm used for their literature research. For the interest of transparency, it would be helpful, however, if they could explain the underlying principles for their search strategy in a few sentences. | Thank you for your suggestion. We have added the following section explaining the underlying principles for the search strategy in the ‘information sources and search strategy’ section:  ‘The search strategy was developed using phrases and keywords of “psychological interventions related to depression”, “children and adolescent populations” and “cross-cultural adaptation”. These terms were connected using Boolean operators and truncations where appropriate.’ |
| Results: |  |
| 6.Table 1: Consistent use of bold letters for headings; consistent, equal structure of table cells (e.g. column „setting and sample“ – please provide information on diagnoses and country of implementation for every study – if unknown, this can be noted accordingly); about adding a column mentioning the kind of cross-cultural adaptation process used by each study should be thought | Thank you for highlighting this. We have made the changes in Table 1.  We have added information about diagnosis for the study by Morsette (2009).  Country of implementation: Bernal et al. (2019), Rosselló, Bernal & Rivera-Medina (2008) , Morsette (2009), Duarté -Vélez and Bernal & Bonilla (2010).  However, we have not added a column mentioning the kind of cross-cultural adaptation process used by each study because adding more columns will make the table too busy and difficult to follow. This information has been presented separately in Figure 2. |
| 7.Table 3 a-c: These tables all are a bit too confusing for the reader. It should be visible on first sight, which numbers are representing levels of anxiety and which levels of depression. Also, significant changes (pre vs. post as well as treatment- vs. control-group) should be detectable directly for the reader. The column „change reported“(3a)/“significance“ (3b) is very word-heavy. Table 3c can be omitted completely as it provides almost exclusively text content which can be presented within the article’s written results just as well. | Thank you for your suggestion. We have now moved Tables 3 a-c to a supplementary file. Owing to this, we felt it important to retain Table 3c for consistency. Therefore, we present the following: Table 3(a) for reporting RCTs, 3(b) for reporting non-RCTs and 3(c) for reporting case studies.  Where possible we have condensed the information presented in the tables and have presented all anxiety measures in italics to make it easier for the reader to identify the different measures. |
| 8.Figure 2: The benefit from this illustration is arguable as the authors desribe the shown adaptation processes in detail within the results. | Thank you for your suggestion. As this Figure provides an idea of cultural processes used in the included studies at a glance, we felt it important to retain this Figure for readers who prefer a visual presentation of information. |
| 9.In general, the information provided within the tables 3 a-c are repetitive compared to the written results, to a great extend. Due to the large methodological heterogenity of the reviewed studies, the authors chose a narrative synthesis for the presentation of the studies‘ results. However, an abbreviated and focused display of either the written results or the table contents is advisable. | As previously mentioned, we have now presented Tables 3 (a-c) within a supplementary file. This can be viewed by readers if they prefer to see results presented in a tabulated format. We have however condensed the information presented within these tables. |
| 10.p. 23: The authors describe having consulted various stakeholders for discussing their preliminary review findings. From the information shared in the current article, it isn’t clear, however, whether the discussion followed any kind of structured frame or was intentionally kept open. Furthermore, the discussion’s goal and thus the benefit for the current article doesn’t really stand out, so far. Concerning the last paragraph on p. 23, it’s not recognizable whether the authors reproduce the stakeholders‘ points or summarize their own thoughts (which would be misplaced within the results). | Thank you for your suggestion. We have now added some additional sentences to the text:  ‘Some questions were open-ended, for instance asking participants for their thoughts about the findings from the existing research, whilst others were more focused’. |
| Discussion: |  |
| 11.As mentioned above, the discussion’s focus is orientated differently than indicated by abstract and introduction. If the study aims to access the effectiveness of culturally adapted psychological interventions, then the authors have to allow way more space for this topic and discuss their findings critically (e.g. efficacy pre/post, efficacy treatment/non-treatment, (dis-)advantages of different types of control groups, etc.). | As per our response to comment number 4, our updated objectives now correspond to what is presented in the discussion. |
| The authors‘ remarks concerning the benefits of a participatory approach when designing culturally adapted interventions was enlightening. However, the initial observation that „bottom-up“ interventions tend to be more effective than „top-down“ interventions cannot be derived from the results that are presented. Similar applies to the conclusion about emic and etic dimensions by Rosselló and colleagues (p.27), which isn’t mentioned in the article before and is accepted as fact by the authors without further discussion or referencing other supporting literature | Thank you for your suggestion. We have now added the following section relating to the participatory approach:  ‘However, as a comparatively new area of research, further well-designed studies are needed to show the effectiveness of these processes’.  We have also updated the e following sentences about emic and etic dimensions:  ‘This concept of etic and emic norms however only appeared in this study and was only taken so far. As a comparatively new area of research, further work is needed to uncover which elements fall into which category’. |
| Results of the stakeholders discussion (if it shall remain within the article) aren’t discussed at all. | We have now added sentences based on stakeholder consultation findings presented in the discussion section:  ‘The overall positive picture of cultural adaption drawn from the studies aligns with the viewpoint of the stakeholder group that we consulted. They highlighted that such adaptations have the potential to increase the engagement of the participant in intervention delivery and therefore the intervention’s efficacy. It is worth repeating however the group’s cautioning that such an approach needs to go hand in hand with increasing efforts in public, school and community education to improve awareness and understanding of mental health and to challenge stigma.’ |

Response to academic editor’s comment:

1.The manuscript meets PLOS ONE's style requirements and file naming.

2.We addressed the financial disclosure by stating that: “The authors received no specific funding for this work.”

3. We added captions for our Supporting Information files at the end of your manuscript, and update any in-text citations to match accordingly.
